# Supplementary material for: Selective coactivation of α7- and α4β2-nicotinic acetylcholine receptors reverses beta-amyloid–induced synaptic dysfunction
Source: J Biol Chem. 2021 Feb 9;296:100402. doi: 10.1016/j.jbc.2021.100402 (PMC7961090; doi:10.1016/j.jbc.2021.100402)
Supplement: Figures S1 to S3 [file mmc1.docx]

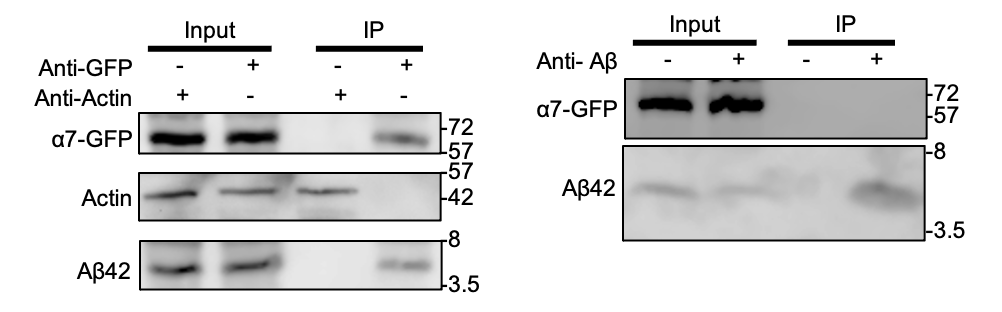


**Supplementary Figure 1. Actin antibody is unable to pull down Aβ42 and α7-nAChRs.** Lysates from cells overexpressing human α7-nAChR-GFP receptors were incubated with 2µM Aβ42 for 18 hours and immunoprecipitated with the anti-actin or anti-GFP antibody. As expected, the anti-actin antibody interacts with actin, while it is unable to pull down α7-nAChRs and Aβ42. Additionally, the anti-GFP antibody was able to interact with α7-nAChRs associated with Aβ42.


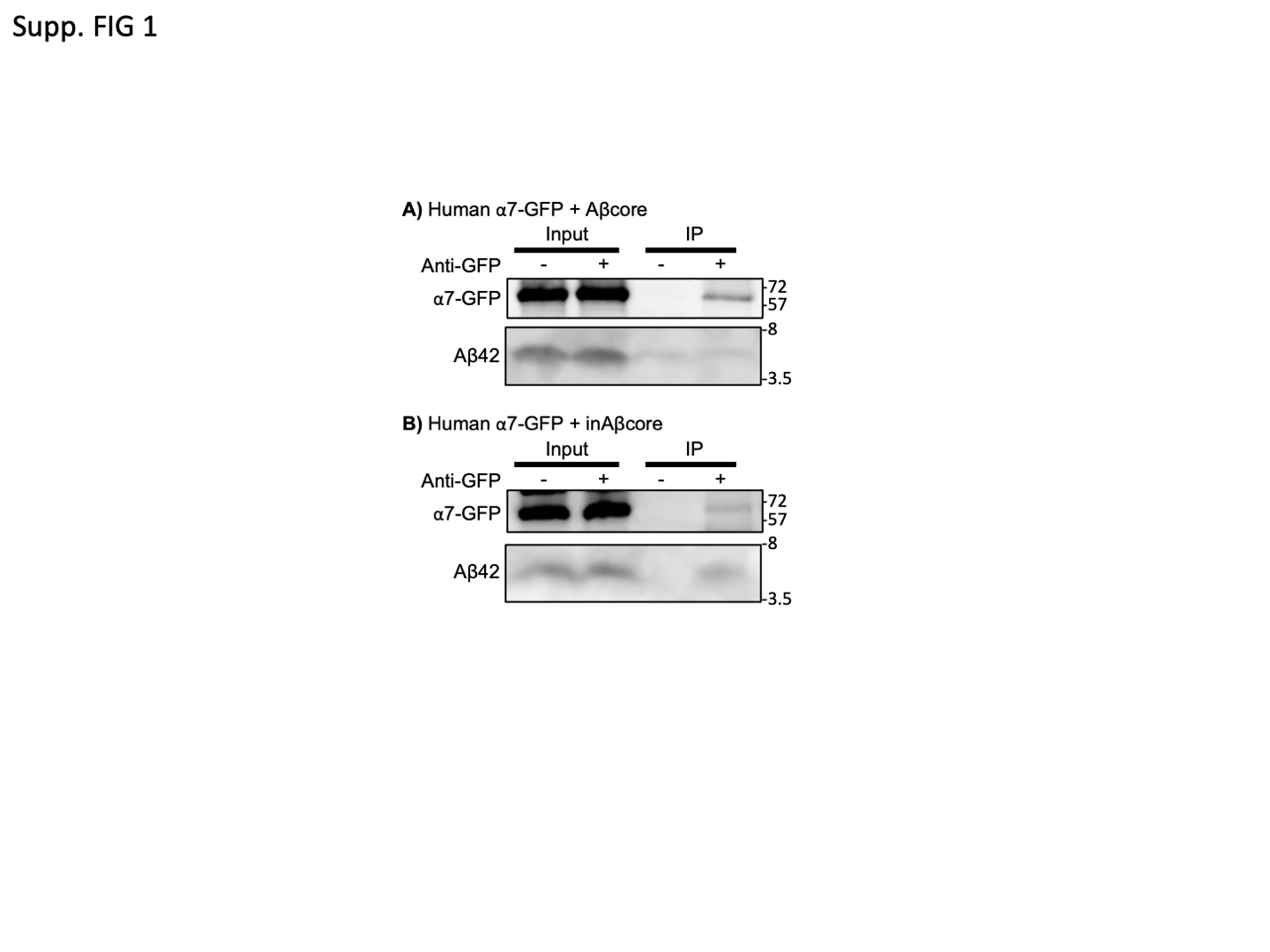


**Supplementary Figure 2. The Aβcore peptide can inhibit the interaction between Aβ42 and α7-nAChRs.** **A)** The active Aβcore peptide (Aβcore) can inhibit the interaction between Aβ42 and α7-nAChRs on co-immunoprecipation. **B)** The inactive Aβcore peptide (inAβcore) has no effect on the interaction between Aβ42 and α7-nAChRs in co-IP.


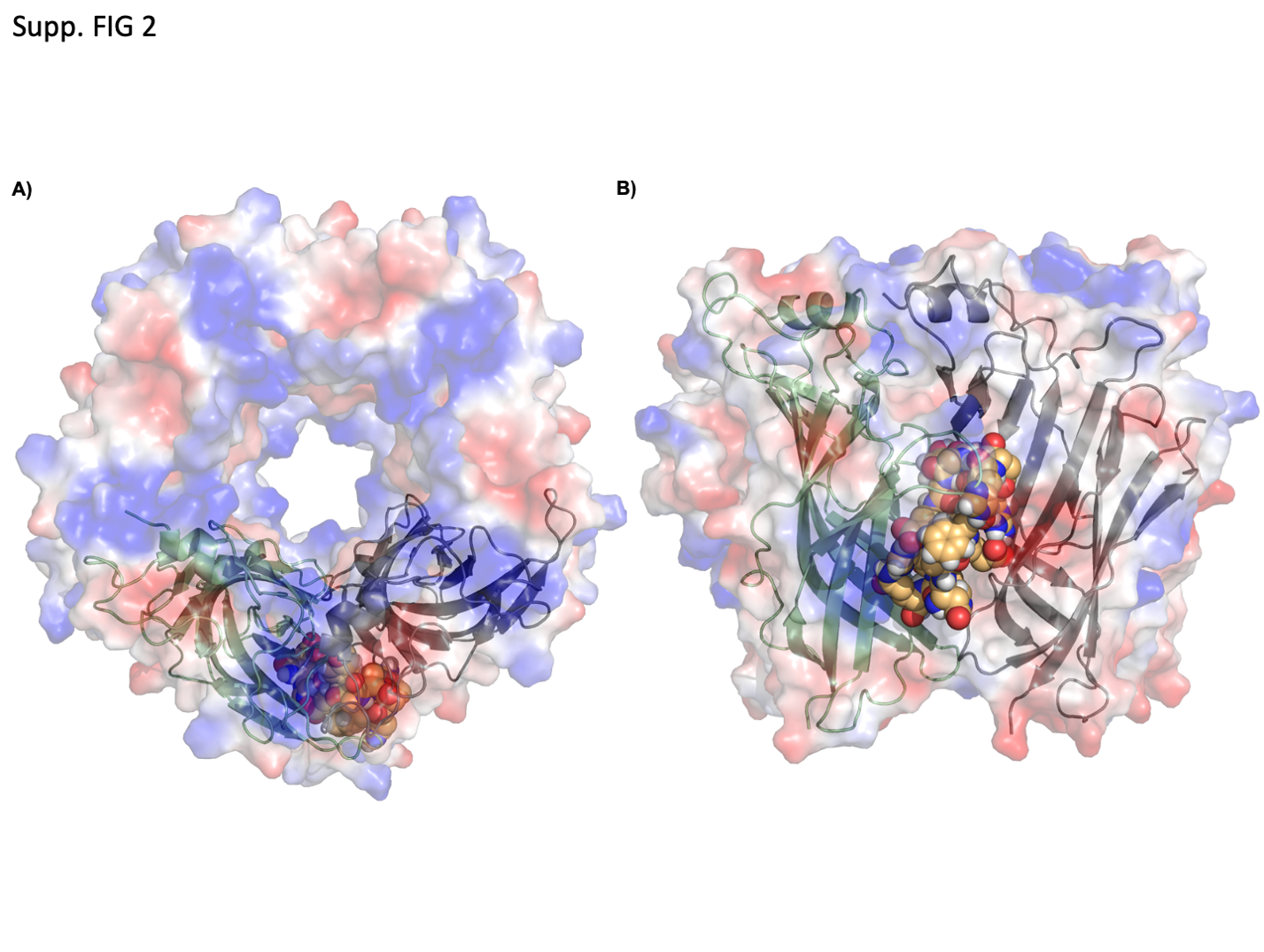


**Supplementary Figure 3.** A docking model between N-terminus of Aβ (1-15 amino acids) and pentameric extracellular domains in acetylcholine-binding protein (AChBP) by using CABS-dock server for flexible protein-peptide docking suggests Aβ is likely to sit between two protomers among pentamers. N-terminus of Aβ is shown as a sphere model within vacuum electrostatic model of extracellular domains in AChBP and only two protomers among pentamers are shown as cartoon drawing in top (A) and side views (B).
